# Vasoresponsiveness in patients with heart failure (VASOR): protocol for a prospective observational study

**DOI:** 10.1186/s13019-019-1014-8

**Published:** 2019-11-21

**Authors:** Marieke E. van Vessem, Saskia L. M. A. Beeres, Rob B. P. de Wilde, René de Vries, Remco R. Berendsen, Evert de Jonge, A. H. Jan Danser, Robert J. M. Klautz, Martin J. Schalij, Meindert Palmen

**Affiliations:** 1 Department of Cardiology, Leiden University Medical Center, PO Box 9600, Leiden, 2300 RC The Netherlands; 2 Department of Cardiothoracic Surgery, Leiden University Medical Center, PO Box 9600, Leiden, 2300 RC The Netherlands; 3 Department of Intensive Care, Leiden University Medical Center, Leiden, the Netherlands; 4 Department of Internal medicine, Erasmus Medical Center, Rotterdam, The Netherlands; 5 Department of Anesthesiology, Leiden University Medical Center, Leiden, the Netherlands

**Keywords:** Vasoplegia, Vasoplegic syndrome, Vasodilatory shock, Heart failure surgery, Vasoresponsiveness, Vasoreactivity

## Abstract

**Background:**

Vasoplegia is a severe complication which may occur after cardiac surgery, particularly in patients with heart failure. It is a result of activation of vasodilator pathways, inactivation of vasoconstrictor pathways and the resistance to vasopressors. However, the precise etiology remains unclear. The aim of the Vasoresponsiveness in patients with heart failure (VASOR) study is to objectify and characterize the altered vasoresponsiveness in patients with heart failure, before, during and after heart failure surgery and to identify the etiological factors involved.

**Methods:**

This is a prospective, observational study conducted at Leiden University Medical Center. Patients with and patients without heart failure undergoing cardiac surgery on cardiopulmonary bypass are enrolled. The study is divided in two inclusion phases. During phase 1, 18 patients with and 18 patients without heart failure are enrolled. The vascular reactivity in response to a vasoconstrictor (phenylephrine) and a vasodilator (nitroglycerin) is assessed in vivo on different timepoints. The response to phenylephrine is assessed on t1 (before induction), t2 (before induction, after start of cardiotropic drugs and/or vasopressors), t3 (after induction), t4 (15 min after cessation of cardiopulmonary bypass) and t5 (1 day post-operatively). The response to nitroglycerin is assessed on t1 and t5. Furthermore, a sample of pre-pericardial fat tissue, containing resistance arteries, is collected intraoperatively. The ex vivo vascular reactivity is assessed by constructing concentrations response curves to various vasoactive substances using isolated resistance arteries. Next, expression of signaling proteins and receptors is assessed using immunohistochemistry and mRNA analysis. Furthermore, the groups are compared with respect to levels of organic compounds that can influence the cardiovascular system (e.g. copeptin, (nor)epinephrine, ANP, BNP, NTproBNP, angiotensin II, cortisol, aldosterone, renin and VMA levels). During inclusion phase 2, only the ex vivo vascular reactivity test is performed in patients with (*N* = 12) and without heart failure (N = 12).

**Discussion:**

Understanding the difference in vascular responsiveness between patients with and without heart failure in detail, might yield therapeutic options or development of preventive strategies for vasoplegia, leading to safer surgical interventions and improvement in outcome.

**Trial registration:**

The Netherlands Trial Register (NTR), NTR5647. Registered 26 January 2016.

## Background

The incidence and prevalence of chronic heart failure is increasing. Despite the expansion of therapeutic options, including the development of new pharmacological therapies and cardiological interventions, overall survival and quality-of-life remains poor [1]. When optimal medical therapy and cardiological interventions have failed to improve a patient’s condition, surgical intervention may be a valid option in order to improve cardiac function. Surgical treatment of end-stage chronic heart failure encompasses different treatment modalities like surgical revascularization of ischemic territories using coronary artery bypass grafting (CABG), alleviating functional mitral valve insufficiency (using restrictive mitral annuloplasty) and reconstructing left ventricular geometry and thereby improving contractility in patients that suffered from a large myocardial infarction resulting in a scarred and dilated left ventricle. Ultimately, left ventricular function can be replaced by performing orthotopic heart transplantation or by implantation of a left ventricular assist device (LVAD). These surgical options have improved clinical outcome [2–4]. Unfortunately, heart failure surgery is associated with an increased risk on vasoplegia, also named vasodilatory shock [5]. This syndrome is characterized by hypotension and the continuous need of vasopressors, despite a normal or high cardiac index. The incidence of vasoplegia ranges from 11 to 31% in patients undergoing heart failure surgery [5–9]. The prognosis of vasoplegia is poor. Prolonged hypotension and the accompanying hypoperfusion lead to end-organ dysfunction and is associated with an increased morbidity. An earlier study showed that the 90-day survival rate after heart failure surgery is decreased in vasoplegic patients compared with non-vasoplegic patients (71% vs 91%, *P* < 0.001) [8].

**Fig. 1:**
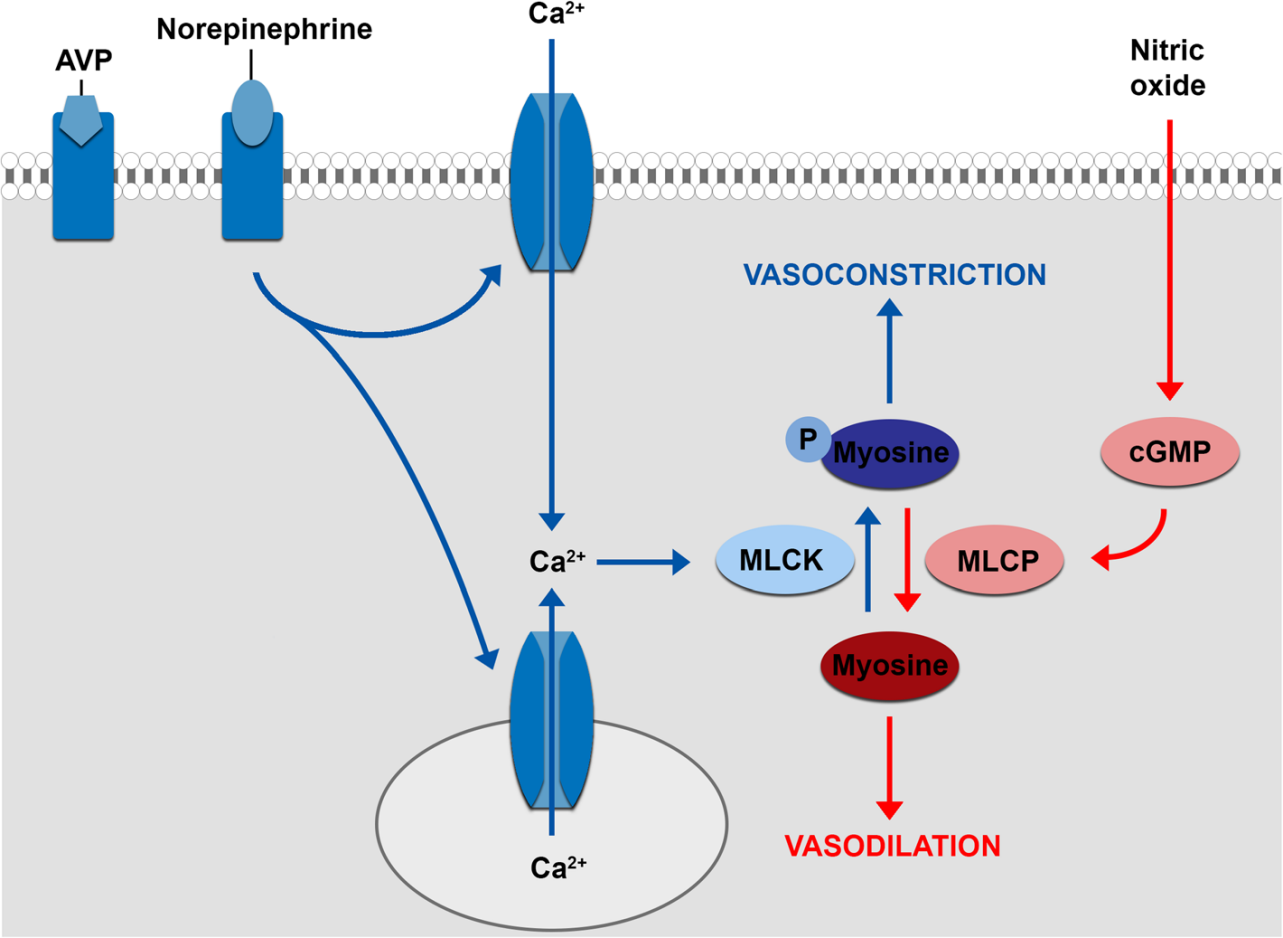
Regulation of vascular smooth muscle tone. Binding of arginine vasopressin and norepinephrine to their receptor on the vascular smooth muscle cell surface results in an increase of the calcium concentration in the cytosol, thereby activating myosin light chain, leading to vasoconstriction. Vasodilators (e.g. nitric oxide, atrial natriuretic peptide) deactivate myosin light chain, introducing vasodilatation. AVP, arginine vasopressin; Ca^2+^, calcium ion, cGMP, cyclic guanosine monophosphate; MLCK, myosin light chain kinase; MLCP, myosin light chain phosphatase. Adapted from Landry and Oliver [10]

Vasoplegia is a result of failure of the vascular smooth muscle cells to constrict to normal endogenous and exogenous stimuli. Normally, a vascular smooth muscle cell constricts due to binding of a ligand (e.g. arginine vasopressin or norepinephrine) to a receptor on the vascular smooth muscle cell surface (Fig. 1). This activates a signal transduction pathway, resulting in an increase of the calcium concentration in the cytosol due to release of intracellular calcium and an influx of extracellular calcium through voltage-gated calcium channels. Binding of calcium to calmodulin leads to phosphorylation of myosin light chain kinase, which activates myosin light chain, leading to vasoconstriction. In contrast, vasodilators (e.g. nitric oxide, atrial natriuretic peptide) increase cyclic guanosine monophosphate (cGMP) concentrations in the vascular smooth muscle cell. This leads to the activation of myosin light chain phosphatase, which deactivates myosin light chain, introducing vasodilatation. The mechanism that causes vasoplegia is thought to be multifactorial. It seems to involve activation of vasodilator pathways and inactivation of vasoconstrictor pathways resulting in a resistance to vasopressors, but the precise etiology remains subject of debate. Vasodilatory shock due to sepsis is the most studied etiology, but it is likely that the pathophysiological mechanisms differ depending on the underlying etiology. However, Landry and Oliver [10] propose three mechanisms that contribute to all types of vasodilatory shock.

**Fig. 2:**
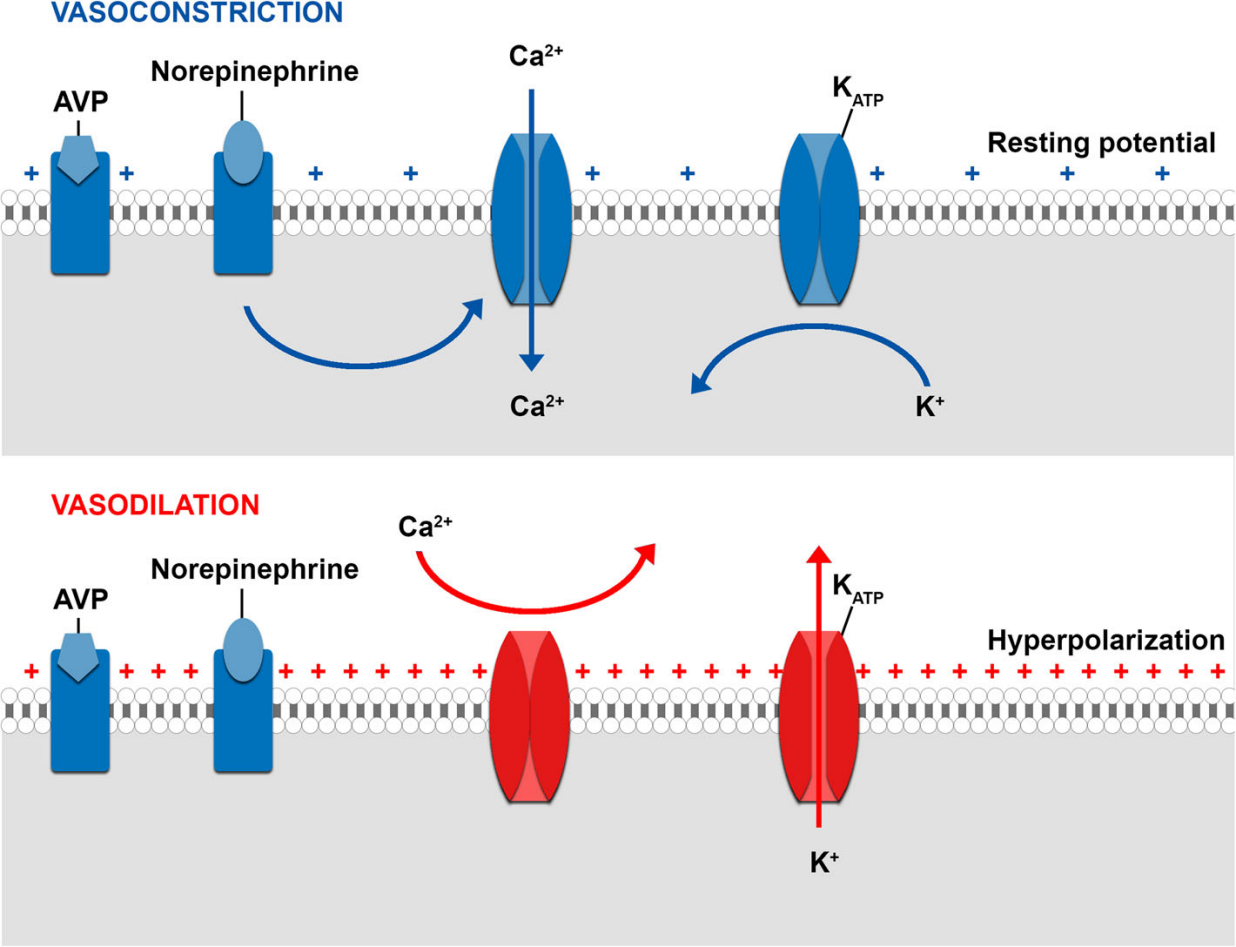
Influence of the K_ATP_ channel on the vascular smooth muscle tone. Closing of K_ATP_ channels leads to depolarization of the vascular smooth muscle cell, thereby opening the voltage gated calcium channels and causing vasoconstriction. Opening of the K_ATP_ channels leads to an efflux of potassium, thereby hyperpolarizing the plasma membrane, causing the voltage gated calcium channels to close, which results in vasodilation. AVP, arginine vasopressin; Ca^2+^, calcium ion; K^+^, potassium ion. Adapted from Landry and Oliver [10]

1. Activation of adenosine triphosphate (ATP) dependent potassium channels (K_ATP_) on the vascular smooth muscle cell.
  When the vascular smooth muscle cell depolarizes, voltage gated calcium channels open, thereby increasing the calcium concentration in the cytosol, causing vasoconstriction. In contrast, hyperpolarization closes the channel, leading to relaxation. K_ATP_ channels influence the membrane potential (Figs. 2 and 3). Opening leads to an efflux of potassium, thereby hyperpolarizing the plasma membrane, causing the voltage gated calcium channels to close. Under normal circumstances K_ATP_ channels are closed, but they open when intracellular ATP concentration is low and when lactate and hydrogen ion concentrations are high, like during hypoxic and increased metabolic states. Atrial natriuretic peptide, calcitonin gene-related peptide, adenosine and increased nitric oxide concentrations (indirectly) may open the channel as well.

**Fig. 3:**
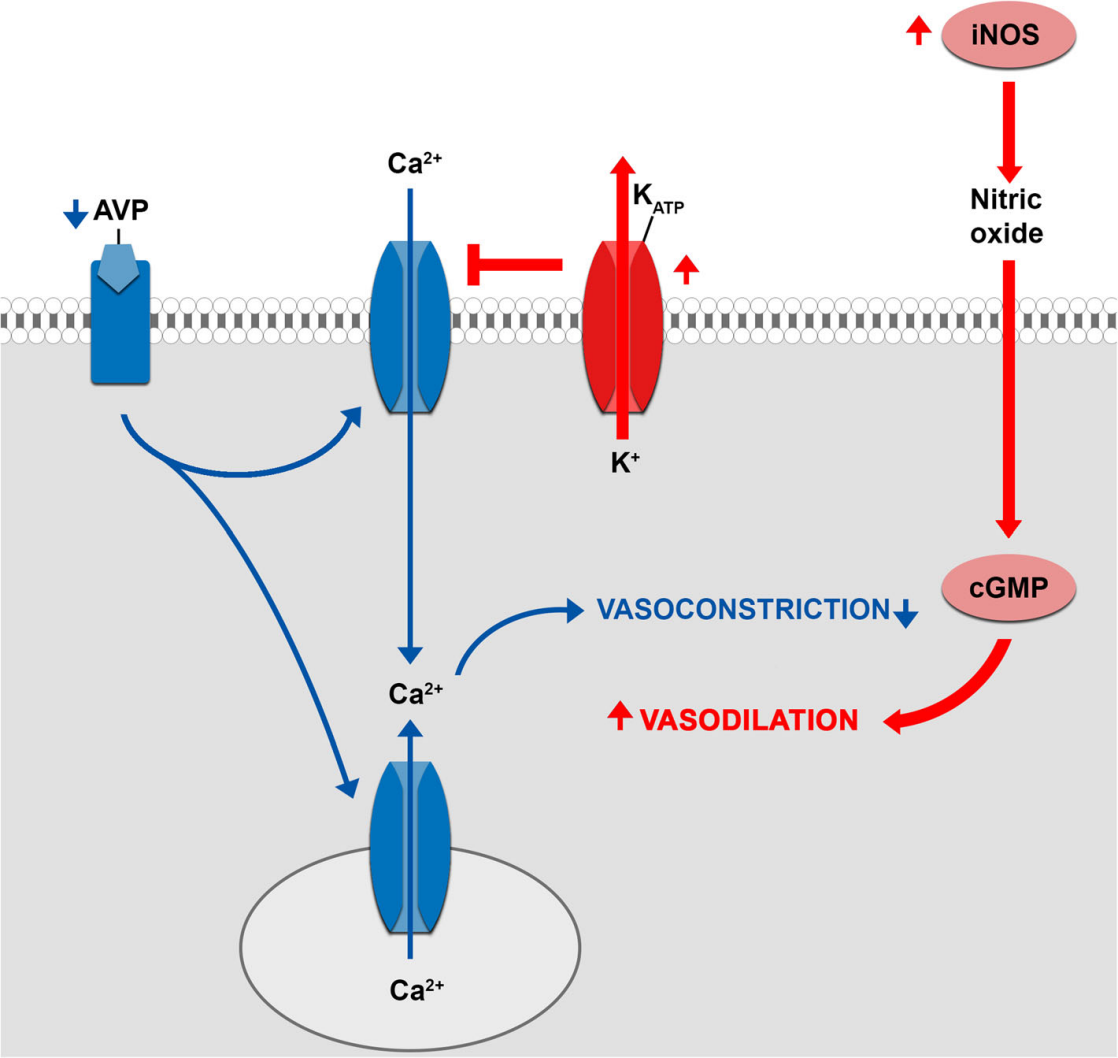
Summary of the three mechanisms contributing to vasodilatory shock: Activation of adenosine triphosphate (ATP) dependent potassium channels (K_ATP_), activation of inducible nitric oxide synthase (iNOS) and arginine vasopressin (AVP) deficiency. Adapted from Landry and Oliver [10]
2. Activation of inducible nitric oxide synthase (iNOS).
  iNOS (NOS2), is one of the three isoforms of nitric oxide synthase. The other forms are neuronal nitric oxide synthase (nNOS or NOS1) and endothelial nitric oxide synthase (eNOS or NOS3). The synthases are responsible for the production of nitric oxide. Therefore, activation of iNOS leads to increased vasodilatation (see Fig. 3). Accordingly, the use of methylene blue, a cGMP inhibitor, seems to be effective for the treatment of vasoplegic syndrome [11].
3. Deficiency of arginine vasopressin (AVP).
  The binding of AVP to the vascular smooth muscle cell leads to vasoconstriction. Accordingly, a deficiency in AVP leads to a reduced ability of the vascular smooth muscle cell to constrict (see Fig. 3). The role of AVP in vasoplegic shock is confirmed by Colson et al. [12] who showed that vasoplegic patients have higher preoperative copeptin (a precursor of AVP) plasma concentrations, but lower AVP concentration postoperatively.

Besides these mechanisms, we postulate that other characteristics of heart failure patients make them more prone to develop vasoplegia. For instance, the chronic endogenous adrenergic (over)stimulation leads to downregulation and desensitization of myocardial β_1_-adrenergic receptors and desensitization β_2_-adrenergic receptors in heart failure [13]. This continuous adrenergic stimulation also seems to result in downregulation and/or desensitization of vascular α_1_-adrenoreceptors [14], leading to an altered responsiveness of the vascular system of heart failure patients. We hypothesize that the balance of the vascular system of patients with heart failure is fragile and therefore could easily be disturbed by the systemic inflammatory response (SIRS) reaction caused by the cardiopulmonary bypass and surgical trauma [15], making these patients more prone to develop vasoplegia. Furthermore, the sympathetic activation is likely to be related to the proinflammatory state of a heart failure patient [16]. In addition, the medication that is prescribed to heart failure patients (e.g. beta blockers, ACE inhibitors, angiotensin receptor blockers, diuretics) influences the hemodynamics as well and could contribute to the risk on vasoplegia. Most probably all the above described factors may play a role in the development of vasoplegia after heart failure surgery, but this has never been proven in patients.

## Methods

The Standard Protocol Items: Recommendations for Interventional Trials (SPIRIT) checklist is provided in Additional file 1.

### Study design

**Fig. 4:**
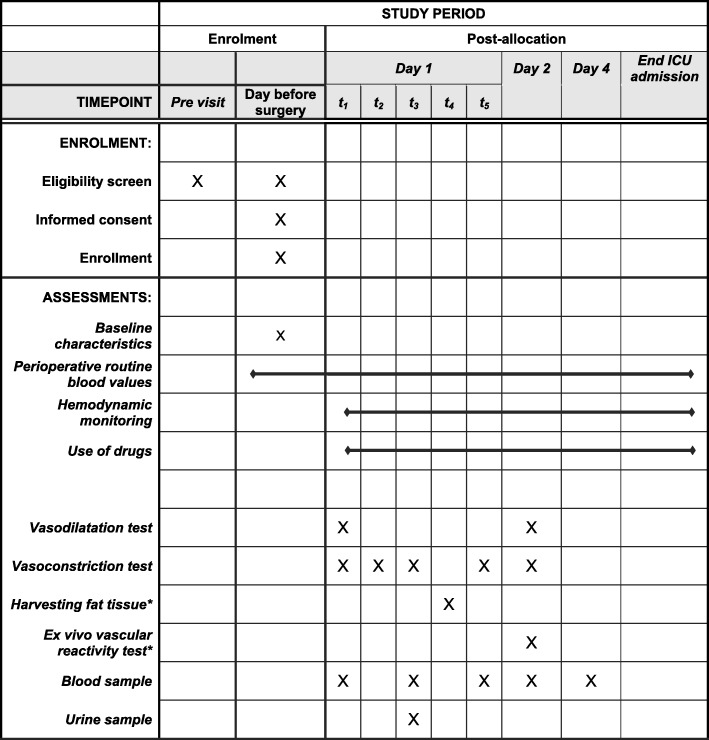
Schedule of enrolment and assessments (SPIRIT statement). * During phase 2, only fat tissue will be harvested and the ex vivo vascular reactivity test will be performed. t_1_, before induction; t_2_, before induction, after start of cardiotropic drugs and/or vasopressors when necessary; t_3_, after induction; t_4_, before the cardiopulmonary bypass is connected; t_5_, 15 min after cessation of cardiopulmonary bypass. Adapted from Landry and Oliver [10]

The aim of the current study is to objectify and characterize the altered vasoresponsiveness in patients with heart failure, before, during and after heart failure surgery and to identify the etiological factors involved. This is a prospective, observational study, conducted at the Leiden University Medical Center. Patients with heart failure will be compared with patients without heart failure. Figure 4 shows a schematic overview of the study (SPIRIT statement). The study is divided in two inclusion phases. The protocol of the patients who are included in phase 1 consists of several in vivo vascular reactivity tests, an ex vivo vascular reactivity test and blood and urine sample analysis. In patients included in phase 2, only the ex vivo vascular reactivity test is performed.

### Participants

Two researchers (MV and MP) screen patients scheduled for elective or urgent cardiac surgery on cardiopulmonary bypass for eligibility. Patients are recruited for either the heart failure group or the non-heart failure group. Heart failure is defined according to the European Society of Cardiology guidelines [17]. In order to be eligible for inclusion in the heart failure group, patients must meet all of the following inclusion criteria: 1) Diagnosed with heart failure and 2) LVEF ≤35%. Patients that are included in the non-heart failure group are 1) not diagnosed with heart failure and 2) have a LVEF > 50%. In addition, patients are selected according to the expected cardiopulmonary bypass and estimated aortic cross clamp duration.

A patients who meets any of the following criteria is excluded from participation in this study: 1) age < 18 years; 2) incapacitated adults; 3) emergency operation; 4) patients in need of moderate of high dosages of intravenous inotropic support (> 4 gamma dobutamine or dopamine), vasopressin and/or mechanical support; 5) patients with aortic valve insufficiency > grade 1; 6) patients using a daily dosage of nitrates or 7) α-adrenergic blockers; and 8) patients not willing to sign the consent form. All included patients receive a subject identification code starting at 1001, up to 1060.

### Clinical parameters

Baseline characteristics (including age, gender, EuroSCORE, comorbidity, medication), perioperative routine blood values, use of (vasoactive) drugs (e.g. phenylephrine, norepinephrine, epinephrine, dopamine, dobutamine, milrinone), hemodynamic parameters and transfusion products are registered in the hospital’s electronic patient information system.

### Anesthetics and hemodynamic monitoring

Anesthetics are given according to a standard protocol. Patients are anesthetized with target-controlled infusion of propofol and remifentanil or sufentanil. Bispectral index monitoring is used to guide the anesthetic dosing. Ketamine and sevoflurane are not used.

Before induction all patients will receive an arterial line for invasive monitoring of blood pressure and blood sampling. A central venous catheter is inserted in the internal jugular vein and a flow-directed balloon-tipped pulmonary artery catheter is introduced after induction. The pressure transducers of the arterial catheter and central venous catheter are connected to separate M1006A invasive blood pressure modules (Hewlett-Packard-medical-products-group, Andover, MA. USA) for optimal data recording at a frequency of 100 Hz and the resolution 0.2 mmHg.

Three different systems are used for hemodynamic measurements. 1) The FloTrac-sensor of the radial artery catheter is connected to a Vigileo system (Edwards LifeSciences, Irvine, CA, USA). The system uses the arterial pressure waveform and patient characteristics (height, weight, age and sex) to estimate cardiac output/stroke volume. The value of central venous pressure was entered into the Vigileo-monitor in order to calculate systemic vascular resistance (SVR). 2) The pulmonary artery catheter is connected to a Vigilance-II monitor (Edwards LifeSciences, Irvine, CA, USA). The patient characteristics (height, weight, age) are entered in the monitor and used for the algorithm. 3) PulseCO™ software (LiDCO, London, UK) is used for measurement of hemodynamic variables as arterial blood pressure, cardiac output/stroke volume, pulse pressure variation, stroke volume variation and after entering central venous pressure, SVR. The system uses arterial waveform and patient characteristics (age, height, weight, and value of hemoglobin). The cardiac output derived from the Vigilance-II monitor, is used to calibrate the cardiac output as measured by PulseCO. Software of the LiDCOplus (LiDCOviewSE, LiDCO Ltd., London, UK) is used for off-line analysis of the vasoreactivity tests. The decision to evaluate hemodynamic variables with pulse contour analysis lay in the character of these monitoring systems. FloTrac/Vigileo and PulseCO, providing a quick response time to medication induced vaso(motor)reactivity by beat to beat analysis of arterial blood pressure.

### Surgical procedures

Main procedures that are performed are mitral valve plasty and aorta surgery. In the heart failure group left ventricular reconstruction and left ventricular assist device implantation are performed as well. All surgeries are performed via a midline sternotomy with the use of cardiopulmonary bypass with antegrade warm blood cardioplegia.

### In vivo vascular reactivity test

The vascular reactivity in response to a vasoconstrictor (phenylephrine, an α_1_-adrenoreceptor agonist) and vasodilator (nitroglycerin) is assessed for all patients in phase 1 of the study. During the vasoconstriction test, a bolus of 2 μg/kg phenylephrine is administered intravenously, after which the effect on SVR and MAP is registered. The test is performed 5 times: t1) before induction; t2) before induction, after start of cardiotropic drugs and/or vasopressors (e.g. dobutamine, milrinone, norepinephrine) when necessary; t3) after induction; t4) 15 min after cessation of cardiopulmonary bypass; and t5) on the first postoperative day. The timepoints reflect different stages perioperatively, during which the hemodynamic situation changes and that are present in all procedures that are included in this study.

For the vasodilation test a bolus of 10 μg nitroglycerin is given intravenously. The effect on SVR and MAP are registered. Dosages are increased to 20 μg, 40 μg and 60 μg until a drop of 10% in MAP is reached. The vasodilation test is performed twice: t1) before induction and t5) 1 day post-operatively. The test is only performed at these timepoints since we anticipate that in most heart failure patients the test cannot be performed intraoperatively due to the hemodynamic effect. Before the test is started, the patient needs to be in supine rest for at least 10 min.

Both the vasoconstriction and the vasodilation test will only be performed when the clinical condition of the patient allows an increase or drop in MAP.

### Ex vivo vascular reactivity tests

This test will be conducted in both phase 1 as phase 2 of the study. A sample of pre-pericardial fat tissue, containing resistance arteries, is collected before the cardiopulmonary bypass is connected. The tissue is directly preserved in Krebs-Henseleit buffer and transferred in a cooled box (± 4 °C) to the Erasmus Medical Center. Here, the tissue is stored overnight at 4 °C. The next morning, the arterioles are isolated, cut into ring segments of ±2 mm length and mounted in a Mulvany myograph. The 6 ml organ baths contain gassed Krebs-Henseleit buffer at 37 °C. The tension on the segments is normalized to 90% of the estimated diameter at 100 mmHg of effective transmural pressure. After a 30-min stabilization period, the maximal contractile response is determined by exposing the vessels to 30 and 100 mmol/L of potassium chloride. The following concentration-response curves (CRCs) are constructed: 1) phenylephrine (1 to 100 nmol/L); 2) vasopressin (0.1 to 300 nmol/L); 3) sodium nitroprusside (SNP) (1–100 μmol/L); and 4) bradykinin (0.1 to 1000 nmol/L). bradykinin CRCs are constructed in the absence or presence of 5) 1H-[1,2,4]oxadiazolo[4,3-a]quinoxalin-1-one (ODQ) (10 μmol/L, after concentration response curve 0.1 to 10 μmol/L) and 6) N^G^-nitro-L-arginine methyl ester (L-NAME) (100 μmol/L, after CRC 1 to 100 μmol/L). SNP and bradykinin induced relaxation was assessed after preconstriction with U46619 (10 to 30 nmol/L, aiming for 70–100% of the 100 mmol/L potassium chloride contraction).

Another sample of fat tissue is fixed in 4% formaldehyde for 7 days and paraffin-embedded for later determination of activated signaling proteins (e.g. protein kinase C, protein kinase G, protein kinase A) and the expression of receptors (e.g. β_2_, α_1_, V_1a_, AT_1_) using immunohistochemistry. Furthermore, a small segment of arterial tissue is snap-frozen in liquid nitrogen and stored at − 80 °C for mRNA analysis. Precise analysis will be guided by the findings of the functional tests.

### Blood and urine sample

Arterial blood samples (one 10 ml ethylenediaminetetraacetic acid (EDTA) tube and one 8.5 ml serum-separating tube) are collected at 5 time points during phase 1 of the study: 1) before induction; 2) after induction; 3) after cardiopulmonary bypass; 4) on day 1 post-operative and 5) on day 3 post-operative. The samples are centrifuged at 1500 g, at 4 °C for 10 min. Plasma and serum are stored in five 500 μl cups at − 80 °C to analyse levels of organic compounds that can influence the cardiovascular system (e.g. norepinephrine, epinephrine, ANP, copeptin, NTproBNP, angiotensin II, cortisol, aldosterone, renin, IL-1, IL-6 and TNF-α).

After an urinary catheter is placed, a urine sample is collected. The sample is stored at − 80 °C until analysis for levels of organic compound that effect the cardiovascular system (e.g. steroids, VMA, angiotensinogen). Precise analysis will be guided by the findings of the functional tests.

### Study parameters

The primary outcome is the change in SVR after phenylephrine administration at baseline.

The secondary outcomes are 1) change in MAP after phenylephrine administration; 2) change in MAP and SVR after nitroglycerin administration; 3) vasoplegia (defined as the continuous need of vasopressors (norepinephrine ≥0.2 μg/kg/min for at least 12 consecutive hours, terlipressin or methylene blue) in combination with a cardiac index ≥2,2 l/min/m2 for at least 12 consecutive hours, starting within the first 3 days post-operatively); 4) Copeptin, norepinephrine, epinephrine, ANP, BNP, NTproBNP, angiotensin II, cortisol, aldosterone, renin and VMA levels; 5) correlation between change in SVR after phenylephrine administration and clinical parameters (duration, amount and maximal concentration of norepinephrine postoperatively).

Ex vivo secondary outcomes are 1) change in vessel diameter in response to vasoactive drugs; 2) activated signaling proteins which are associated with vasoresponsiveness; and 3) receptors (quantity and function) which are associated with vasoresponsiveness.

### Sample size calculation

The primary outcome is change in SVR after phenylephrine administration at baseline. A sample size of 17 in each group will have 90% power to detect a difference in means of 400 dyn·s/cm5 assuming that the common standard deviation is 350 dyn·s/cm5 [14] using a student t-test with a 0.05 two-sided significance level. One extra patient is included in each group to compensate for possible loss of data due to failing of the test, so 36 patients are included in total in phase 1 of the study. In phase 2, 24 extra patients will be included.

### Statistical analysis

All data of the in vivo and ex vivo tests are analyzed by a researcher (respectively RW and RV) blinded for the patient group. Baseline patient characteristics are described using summary statistics. Continuous variables are reported as mean with SD when normal distributed, or as median with interquartile range when appropriate. Differences between groups (heart failure versus no heart failure) are compared using an unpaired Students t-test, or Mann Whitney U-test. Categorical data is reported as numbers and percentages. Fisher’s exact test is used to compare the differences between groups. Pairwise deletion is used to handle missing data. The effect of the vasoreactivity tests is adjusted for the level of used vasoactive medication. The significance level is set at *P* < 0.05. The Statistical Package for the Social Sciences (SPSS, version 22) is used for the statistical analyses.

### Consent

Participation in the study is voluntary and written informed consent is obtained by the investigators (MV and MP). Participants can withdraw their consent at any time. Study findings will be disseminated through peer-reviewed publications.

### Data management

Handling of data complies with the Dutch Personal Data Protection Act (in Dutch: De Wet Bescherming Persoonsgegevens, Wbp). A subject identification code list is used to link data to the patient number of the patient and is stored on a secured computer on the study site. The anonymous dataset is accessible for all investigators of the research team.

### Safety monitoring

A data monitoring committee deemed not to be necessary since it concerns a non-randomized, non-blinded study without serious safety concerns and no follow up. All serious adverse events are reported to the medical ethical committee within 7 days after the responsible investigator has first knowledge of the adverse event. All protocol deviations and adverse events are recorded. The investigators will submit a summary of the progress to the medical ethical committee once a year, including information on numbers of patients included, study progress, (serious) adverse events and amendments.

## Discussion

The incidence and prevalence of chronic heart failure is increasing. It is therefore to be expected that the number of heart failure surgery procedures will increase. Unfortunately, vasoplegia is frequently seen after these procedures and is associated with a poor prognosis. The mechanism that causes vasoplegia is thought to be multifactorial, but the precise etiology remains unclear. This study is designed to improve understanding of these mechanisms.

There are some issues in our study design that need to be noted. First, since the vasoconstriction and the vasodilation test can only be performed when the clinical condition of the patient allows an increase or drop in MAP, we expect that these tests cannot be conducted in all patients. Secondly, baseline vasoreactivity tests are performed uncalibrated since the central venous catheter and the pulmonary artery catheter are introduced after induction to limit the burden for the study patients. Thirdly, collecting a sample of pre-pericardial fat tissue can be difficult in patients who underwent previous cardiac surgery. Furthermore, this tissue is fragile and it can therefore be expected that we will not be able to perform the ex vivo vascular reactivity test in all included patients.

## Conclusion

In summary, this single-center prospective observational study is designed to objectify and characterize the altered vasoresponsiveness in patients with heart failure, before, during and after heart failure surgery and to identify the etiological factors involved. Understanding the difference in vascular responsiveness between patients with and without heart failure in detail, might yield therapeutic options or development of preventive strategies for vasoplegia, leading to safer surgical interventions and improvement in outcome.

## Trial status

The first patient was included on 10 February 2016. To date, 36 patients are enrolled in the study. Recruitment will be completed in 2019.

## Supplementary information

**Additional file 1.** The Standard Protocol Items: Recommendations for Interventional Trials (SPIRIT) checklist.

## Data Availability

The datasets that will be generated during the study will be available from the corresponding author on reasonable request after publication.

## Abbreviations

ATP: Adenosine triphosphate
AVP: Arginine vasopressin
CABG: Coronary artery bypass grafting
cGMP: cyclic guanosine monophosphate
CRC: Concentration-response curve
EDTA: Ethylenediaminetetraacetic acid
eNOS: Endothelial nitric oxide synthase
iNOS: inducible nitric oxide synthase
KATP: Adenosine triphosphate dependent potassium channel
L-NAME: N^G^-nitro-L-arginine methyl ester
LVAD: Left ventricular assist device
nNOS: neuronal nitric oxide synthase
NTR: Netherlands Trial Register
ODQ: 1H-[1,2,4]oxadiazolo[4,3-a]quinoxalin-1-one
SIRS: Systemic inflammatory response
SNP: Sodium nitroprusside
SPSS: STATISTICAL Package for the Social Sciences
SVR: Systemic vascular resistance
Wbp: Dutch Personal Data Protection Act (in Dutch: De Wet Bescherming Persoonsgegevens)
WMO: Medical Research Involving Human Subjects Act (in Dutch: Wet maatschappelijke ondersteuning)
